# Editorial: Mutualistic and antagonistic interactions in the human oral microbiome

**DOI:** 10.3389/fmicb.2025.1731807

**Published:** 2025-11-21

**Authors:** Wen Zhou, Shi Huang, Xiaojing Huang

**Affiliations:** 1 Fujian Key Laboratory of Oral Diseases and Fujian Provincial Engineering Research Center of Oral Biomaterial, School and Hospital of Stomatology, Fujian Medical University, Fuzhou, China; 2 Stomatological Key Lab of Fujian College and University, School and Hospital of Stomatology, Fujian Medical University, Fuzhou, China; 3 Clinical Research Center for Oral Tissue Deficiency Diseases of Fujian Province, School and Hospital of Stomatology, Fujian Medical University, Fuzhou, China; 4 Faculty of Dentistry, The University of Hong Kong, Hong Kong, Hong Kong SAR, China

**Keywords:** oral microorganism, biofilm, oral diseases, systemic diseases, interaction

The human oral cavity is a dynamic ecosystem in which hundreds of microbial species engage in a complex dance of cooperation and competition (Palmer, 2014). These microorganisms significantly impact host health through intricate mutualistic and antagonistic interactions with each other and with host tissues, via mechanisms such as co-adhesion and metabolic exchanges (Marsh and Zaura, 2017). Addressing challenges such as biofilm eradication, bacterial drug resistance, immune evasion, and toxic metabolite production is crucial for preventing oral and systemic diseases, and represents an urgent focus of current research (Baker et al., 2024). This Research Topic presents seven studies, exploring areas like gene regulation, protein modification, vesicle intervention, small molecule modulation, and precise microbial targeting. Collectively, these studies illuminate potential pathogenic mechanisms and pathways linking interactions in the oral microbiome to oral and systemic diseases.

## 1 Genetic-level studies

Numerous genes are implicated in the pathogenicity of oral microbes, such as those encoding glucosyltransferases, acetyltransferases, collagen-binding proteins, and fibronectin. These genes play significant roles in the initiation and progression of pathologies. In addition, specific genes are involved in interactions within the oral microbiome. Advances in genetic detection technologies are rapidly identifying more key genes, facilitating efficient screening for oral and systemic diseases. Fang et al. comprehensively reviewed critical virulence factors of *Streptococcus mutans* (*S. mutans*), including rhamnose-glucose polymers, glucosyltransferases, glucan-binding proteins, and protein antigens, emphasizing the genes encoding these macromolecules. Zhou et al. investigated the *smu_1558c* gene, which encodes a GNAT-family acetyltransferase, revealing its influence on biofilm biomass, microbial growth within biofilms, metabolite composition, and the alteration of biofilm 3D architecture. Their findings underscore the role of acetyltransferases in oral microbial pathogenesis and suggest new avenues for preventing oral and systemic diseases. Through both *in vivo* and *in vitro* studies, Cao et al. revealed that the *LiaS* gene, which belongs to a two-component system, plays a critical role in caries mediated by the interaction between *S. mutans* and *Candida albicans*. Their work has identified the *LiaS* gene as a potential target for treating this complex, infection-induced form of caries. Sang et al. employed a highly sensitive metagenomic method, 2bRAD-M sequencing, in a rat model of oral leukoplakia (OLK). They found oral microbiota shifts precede visible OLK lesions, with increased abundances of *Streptococcus, Glaesserella*, and *Pseudomonas* in biofilms, providing genetic insights that could inform future OLK screening and prevention strategies.

## 2 External modulation of microbes

Membrane vesicles (MVs) are nanoscale bilayer particles secreted by microbes that function as transporters of virulence factors, drug resistance genes, and signaling molecules. They are pivotal in bacterial biofilm formation, immune evasion, and environmental adaptation. According to two reviews in this Research Topic, MVs can deliver toxic macromolecules, establish eDNA-matrix structures, and disseminate drug resistance. These functions provide pathways for oral microbes to disrupt epithelial barriers and bone metabolic homeostasis. This disruption may ultimately lead to systemic diseases. Qiu et al. summarized the latest research on the composition and biological mechanisms of *S. mutans* MVs and their cargo, laying the groundwork for future vaccines, research, and clinical therapies targeting *S. mutans*. Wang et al. reviewed the roles and mechanisms of outer membrane vesicles (OMVs) in periodontitis development, exploring how OMVs transport and release toxic factors such as LPS, proteases, and DNA, contributing to the links between periodontitis and systemic inflammation, thus informing future periodontitis diagnosis and treatment.

Exogenous macromolecules can also regulate the microbial ecosystem. Fluoride has long been proven effective against oral diseases caused by microbial dysbiosis. Ongoing research continues to identify antimicrobial macromolecules, such as chitosan and antimicrobial peptides, for clinical use. Discovering and developing materials with high biocompatibility, efficacy, and specificity is crucial for future treatments of local and systemic diseases. Xu et al., by assessing glucosyltransferase (Gtf) activity, demonstrated that Tannic Acid (TA) significantly inhibits *S. mutans*. They also evaluated biofilm formation and biocompatibility using Crystal Violet staining and CCK-8 assays, proposing TA as a promising candidate for preventing and treating *S. mutans* infections.

## 3 Conclusions and prospects

As the studies in this Research Topic highlight, the oral cavity is more than a passive gateway; it is a dynamic interface where microbial interactions critically influence the host's health. Interactions within the oral microbial ecosystem and with host tissues are vital not only for local oral health but also exert a significant influence on systemic health. Cutting-edge research in this Research Topic suggests that future interventions targeting the complex interactions among oral microbes and between microbes and host tissues—through genetic regulation, MV control, TA intervention, and so on—could effectively manage oral microbiome-derived oral or systemic diseases.

However, challenges remain, including achieving precise regulation, determining optimal dosages, and facilitating clinical translation. Future efforts, such as standardizing multi-species biofilm models, may advance the clinical application of targeted microbial therapies.
